# Upregulated lncRNA SNHG1 contributes to progression of non-small cell lung cancer through inhibition of miR-101-3p and activation of Wnt/β-catenin signaling pathway

**DOI:** 10.18632/oncotarget.14854

**Published:** 2017-01-27

**Authors:** Yun Cui, Fuming Zhang, Chunkai Zhu, Liang Geng, Tongde Tian, Huaimin Liu

**Affiliations:** ^1^ Department of Integrated Chinese and Western Medicine, Henan Provincial Cancer Hospital, Affiliated Tumor Hospital of Zhengzhou University, Zhengzhou, Henan, 450008, China; ^2^ Clinical Laboratory, People's Hospital of Zhengzhou University, Henan Provincial People's Hospital, Zhengzhou, Henan, 450008, China

**Keywords:** non-small cell lung cancer, LncRNA SNHG1, miR-101-3p, SOX9, Wnt/β-catenin signaling pathway

## Abstract

Lung cancer is the most common and aggressive tumor in the world. Long non-coding RNA small nucleolar RNA host gene 1 (lncRNA SNHG1) play critical roles in the progression of cancers. However, the function and underlying mechanism remain unclear in lung cancer. In the current study, we found that expression of SNHG1 was up-regulated in non-small cell lung cancer (NSCLC) tissues and cell lines. NSCLC patients with high SNHG1 expression were significantly correlated with larger tumor size, advanced TNM stage, lymph node metastasis and poor overall survival than patients with low SNHG1 expression. Furthermore, function assays showed that SNHG1 inhibition suppressed NSCLC cell proliferation both *in vitro* and *in vivo*. We also found that miR-101-3p could act as a target of SNHG1 in NSCLC and the inhibition of NSCLC progression induced by SNHG1 knockdown required the activity of miR-101-3p. In addition, we identified that SOX9 acted as a target of miR-101-3p, and SOX9 played the oncogenic role in NSCLC by activating Wnt/β-catenin signaling pathway. Taken together, our study suggested that lncRNA SNHG1 could promote NSCLC progression via miR-101-3p and SOX9. The SNHG1/miR-101-3p/SOX9/Wnt/β-catenin axis regulatory network might provide a potential new therapeutic strategy for lung cancer treatment.

## INTRODUCTION

Lung cancer is the main cause of cancer-related mortality in the world, and about 1.4 million people are diagnosed with lung cancer every year [1, 2]. Two main sub-types of lung cancer are named as non-small cell lung cancer (NSCLC) and small cell lung cancer (SCLC), which account for approximately 80–85% and 15–20% respectively [3]. Although encouraging progress in the diagnosis and treatment for lung cancer, the overall survival rate remains unfavorable [4]. The high mortality is probably related to early metastasis [5]. Thus, a better understanding of the mechanisms involved in the progression of NSCLC and more effective therapeutic approaches are instantly required.

Long non-coding RNAs (lncRNAs) are a class of non-coding RNAs, whose length is more than 200 nucleotides [6]. Recently, many lncRNAs are known to play important roles in cellular development, differentiation, and many other biological processes [7–9]. The dysregulation of lncRNAs have been shown in types of cancer. For example, Zhang et al. showed that upregulation of lncRNA MALAT1 correlated with tumor progression and poor prognosis in clear cell renal cell carcinoma [10]. Li et al. showed that lncRNA BANCR promoted proliferation in malignant melanoma by regulating MAPK pathway activation [11]. Ke et al. reported that knockdown of lncRNA HOTAIR inhibited malignant biological behaviors of human glioma cells via modulation of miR-326 [12]. However, the clinical significance and biological mechanisms of lncRNAs in the progression of NSCLC remain largely unknown.

In the present study, we found that lncRNA SNHG1 was increased in NSCLC tissues and cell lines, high expression of SNHG1 was correlated with larger tumor size, advanced TNM stage, lymph node metastasis and poor overall survival of NSCLC patients. Function analysis showed that SNHG1 inhibition suppressed NSCLC cell proliferation both *in vitro* and *in vivo*. In addition, flow cytometric analysis revealed that the inhibitory effect of SNHG1 inhibition on NSCLC cell proliferation was by causing G1/G0 phases arrest and inducing cell apoptosis. We further identified miR-101-3p as a target for SNHG1. In addition, we suggested that SOX9 acted as a target of miR-101-3p and played the oncogenic role in NSCLC progression by activating Wnt/β-catenin signaling pathway. Taken together, our data suggested that lncRNA SNHG1 could promote NSCLC progression via miR-101-3p and SOX9, and that Wnt/β-catenin signaling pathway was involved in the regulatory mechanism.

## RESULTS

### LncRNA SNHG1 is up-regulated in NSCLC

**Figure 1:**
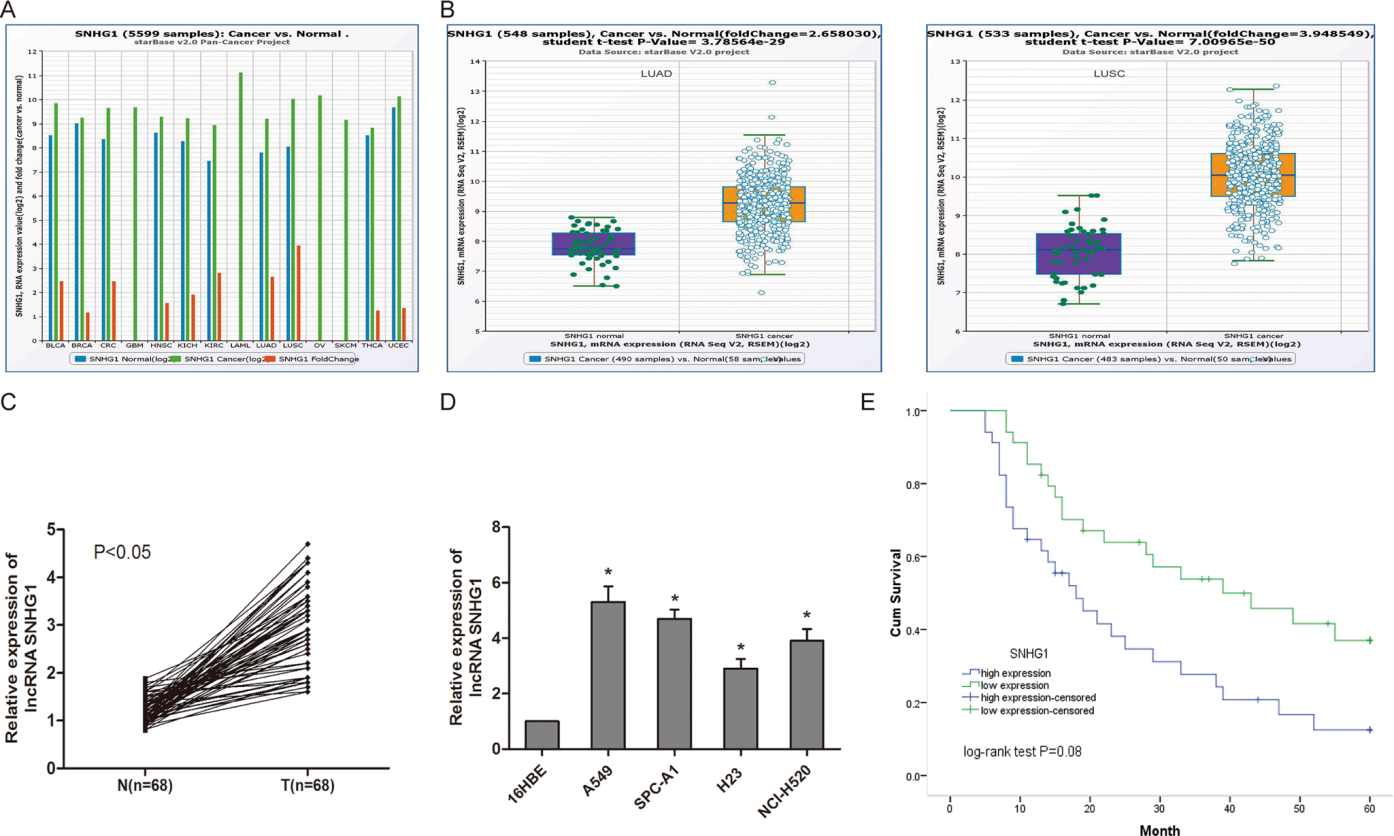
LncRNA SNHG1 was up-regulated in NSCLC (**A**) The expression of SNHG1 among Pan-Cancer including 14 cancer types from The Cancer Genome Atlas (TCGA) Data Portal from starBASE v2.0. (**B**) The expression of SNHG1 in normal or NSCLC (LUAC and LUSC) from TCGA Data Portal. (**C**) The expression of SNHG1 was determined by qRT-PCR in 68 pairs of NSCLC tissues (T) compared with adjacent non-tumour tissues (N). (**D**) The expression of SNHG1 was examined by qRT-PCR in four NSCLC cell lines (A549, SPC-A1, H23 and NCI-H520) and human bronchial epithelial cell line 16HBE. (**E**) The Kaplan-Meier analysis showed that NSCLC patients with high SNHG1 expression had a poor overall survival compared to patients with low SNHG1 expression. LUAC: lung adenocarcinoma; LUSC: lung squamous cell carcinoma. *P < 0.05.

To determine whether lncRNA SNHG1 was associated with progression of NSCLC, we explored SNHG1 expression in TCGA Data Portal from starBASE v2.0. Our findings showed that SNHG1 was higher expression in tumor tissues compared to normal tissues in lung adenocarcinoma (LUAD) and lung squamous cell carcinoma (LUSC) (Figure 1A; *P* < 0.05). SNHG1 expression was also up-regulated in cancer tissues compared to normal counterparts in LUAD and LUSC (Figure 1B; *P* < 0.05). To support this conclusion, we explored SNHG1 expression in NSCLC tissues and cell lines by qRT-PCR. Our findings showed that SNHG1 expression was significantly increased in NSCLC tissues compared to adjacent non-tumor tissues (Figure 1C; *P* < 0.05). In addition, we found that SNHG1 expression was ubiquitously increased in 4 lung cancer cell lines (A549, SPC-A1, H23 and NCI-H520) compared to human bronchial epithelial cell line 16HBE (Figure 1D; *P* < 0.05). Taken together, these data suggested that SNHG1 might play a key role in NSCLC development and progression.

### Up-regulation of lncRNA SNHG1 is correlated with overall survival of NSCLC patients

To further understand the significance of lncRNA SNHG1 up-regulation in NSCLC, we explored the correlation between SNHG1 expression and clinical features of NSCLC patients. We divided the 68 patients into two groups according to the median expression level of SNHG1(high SNHG1 group: SNHG1 expression ratio > median; low SNHG1 group: SNHG1 expression ratio ≤ median). Our data showed that SNHG1 expression in NSCLC tissues was significantly associated with larger tumor size, advanced TNM stage and lymph node metastasis (Table 1, *P* < 0.05). However, there was no significantly correlation of SNHG1 expression with other clinical features such as age, gender, histology and differentiation (Table 1, *P* > 0.05). In addition, Kaplan-Meier analysis and log-rank test were used to evaluate the effects of SNHG1 on overall survival of NSCLC patients. We found that high SNHG1 expression in NSCLC patients had a poor overall survival than NSCLC patients with low SNHG1 expression (Figure 1E; *P* < 0.05).

**Table 1:** Correlation between lncRNA SNHG1 expression and clinicopathological features in NSCLC patients

Parameters	Group	Total	lncRNA SNHG1		*P* value
**Low**	**High**
Gender	Male	41	23	18	**0.215**
	Female	27	11	16	
Age (years)	< 60	30	16	14	**0.625**
	≥ 60	38	18	20	
Tumor size (cm)	< 3 cm	26	17	9	**0.046**
	≥ 3 cm	42	17	25	
Histology	Adenoma	32	18	14	**0.331**
	Squamous	36	16	20	
Differentiation	Well	20	13	7	**0.110**
	Moderate-Poor	48	21	27	
TNM stage	I	31	22	9	**0.002**
	II–III	37	12	25	
Lymph node metastasis	Absence	41	26	15	**0.006**
	Presence	27	8	19	

### LncRNA SNHG1 knockdown inhibits NSCLC cell proliferation *in vitro*

**Figure 2:**
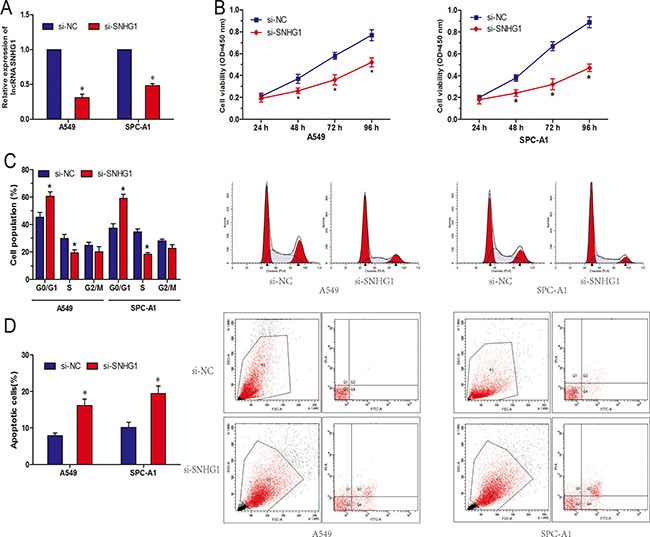
LncRNA SNHG1 inhibition suppressed NSCLC cell proliferation *in vitro* (**A**) Relative expression of SNHG1 after NSCLC cells transfected with si-SNHG1 or si-NC. (**B**) Effect of si-SNHG1 on cell proliferation of A549 and SPC-A1 cells was detected by CCK-8 assay. (**C**) Effect of si-SNHG1 on cell cycle of A549 and SPC-A1 cells was detected by flow cytometry. (**D**) Effect of si-SNHG1 on cell apoptosis of A549 and SPC-A1 cells was detected by flow cytometry. *P < 0.05.

In order to investigate the function of lncRNA SNHG1 in NSCLC, we reduced expression of SNHG1 by transfecting siRNA specifically targeting SNHG1 (si-SNHG1), qRT-PCR showed that SNHG1 expression was obviously downregulated in si-SNHG1 transfected A549 and SPC-A1 cells compared to cells transfected with si-NC (Figure 2A; *P* < 0.05). CCK-8 assay showed that SNHG1 inhibition significantly suppressed cell proliferation both in A549 and SPC-A1 cell lines compared to si-NC group (Figure 2B; *P* < 0.05). Next, flow cytometric analysis was performed to further determine whether the function of SNHG1 on NSCLC cell proliferation was by altering cell-cycle progression or apoptosis. Cell-cycle analysis showed that NSCLC cells transfected with si-SNHG1 were significantly stalled at the G1/G0 phase compared to cells transfected with si-NC (Figure 2C; *P* < 0.05). Cell apoptosis analysis indicated that the apoptosis cells were increased in NSCLC cells transfected with si-SNHG1 compare to si-NC group (Figure 2D; *P* < 0.05). Take together, those findings suggested that knocked-down SNHG1 epression inhibited NSCLC cell proliferation by inhibited cell cycle progression and induced cell apoptosis.

### LncRNA SNHG1 inhibition suppresses NSCLC cell proliferation *in vivo*

**Figure 3:**
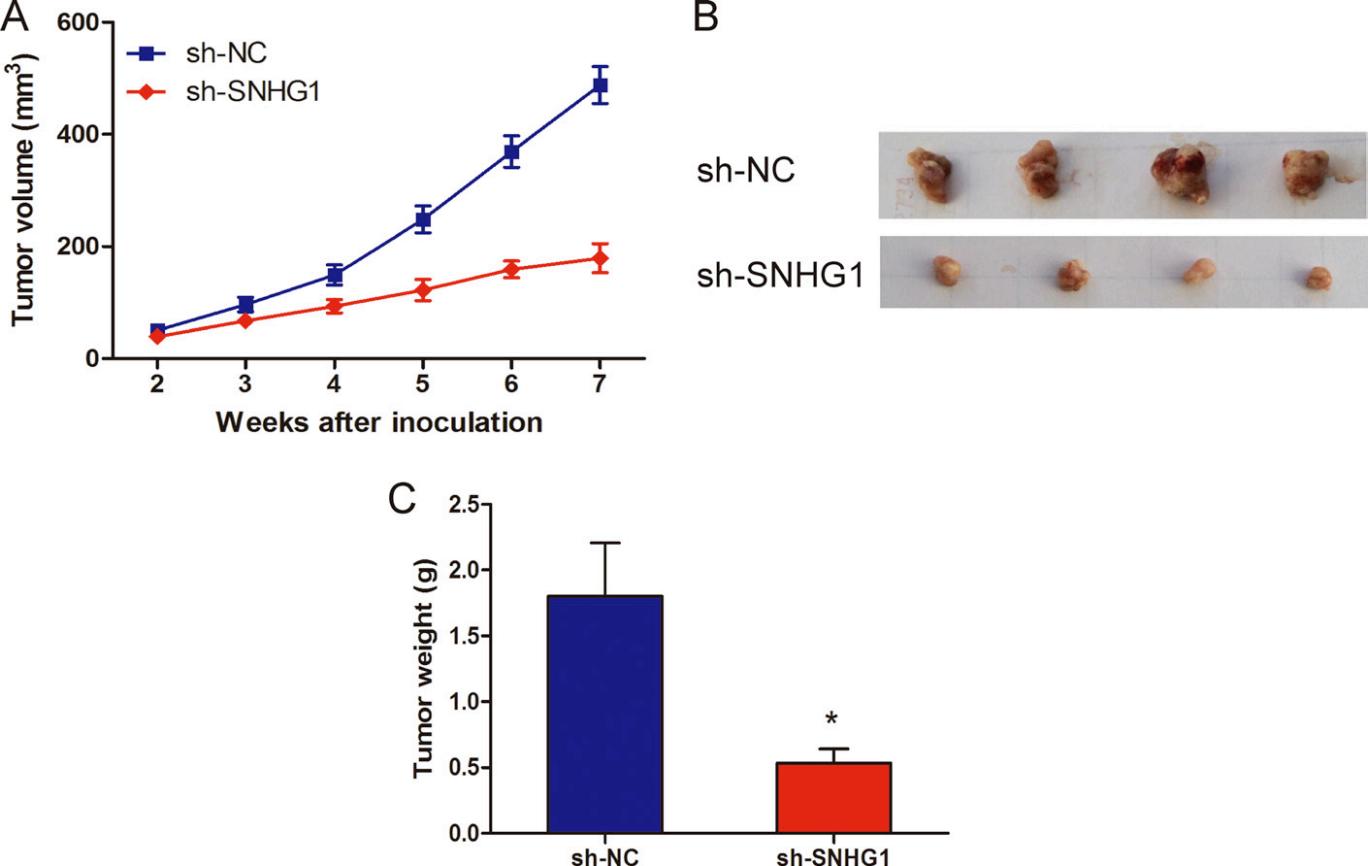
LncRNA SNHG1 depletion inhibited tumor growth *in vivo* (**A**) Tumor growth curves determined after injection of A549 cells transfected with sh-SNHG1 or sh-NC. The tumor volume was calculated every 7 days from 2 to 7 weeks. (**B**) Photographs of tumors excised 49 days after sh-SNHG1 transfected A549 cells injected into nude mice. (**C**) Tumor weight of nude mouse at the end of 49 days. *P < 0.05.

To confirm whether SNHG1 affect NSCLC tumorigenesis, sh-SNHG1 or sh-NC transfected A549 cells were inoculated into nude mice. All mice developed xenograft tumors at the injection site. As shown in Figure 3A and 3B, tumor growth in sh-SNHG1 group was significantly slower than that in sh-NC group (*P* < 0.05). Furthermore, the average tumor weight in the sh-SNHG1 group was obviously lower than in sh-NC group (Figure 3C; *P* < 0.05). These results suggested that knockdown SNHG1 could inhibit proliferation capacity of NSCLC cells *in vivo*.

### MiR-101-3p is an inhibitory target of lncRNA SNHG1

**Figure 4:**
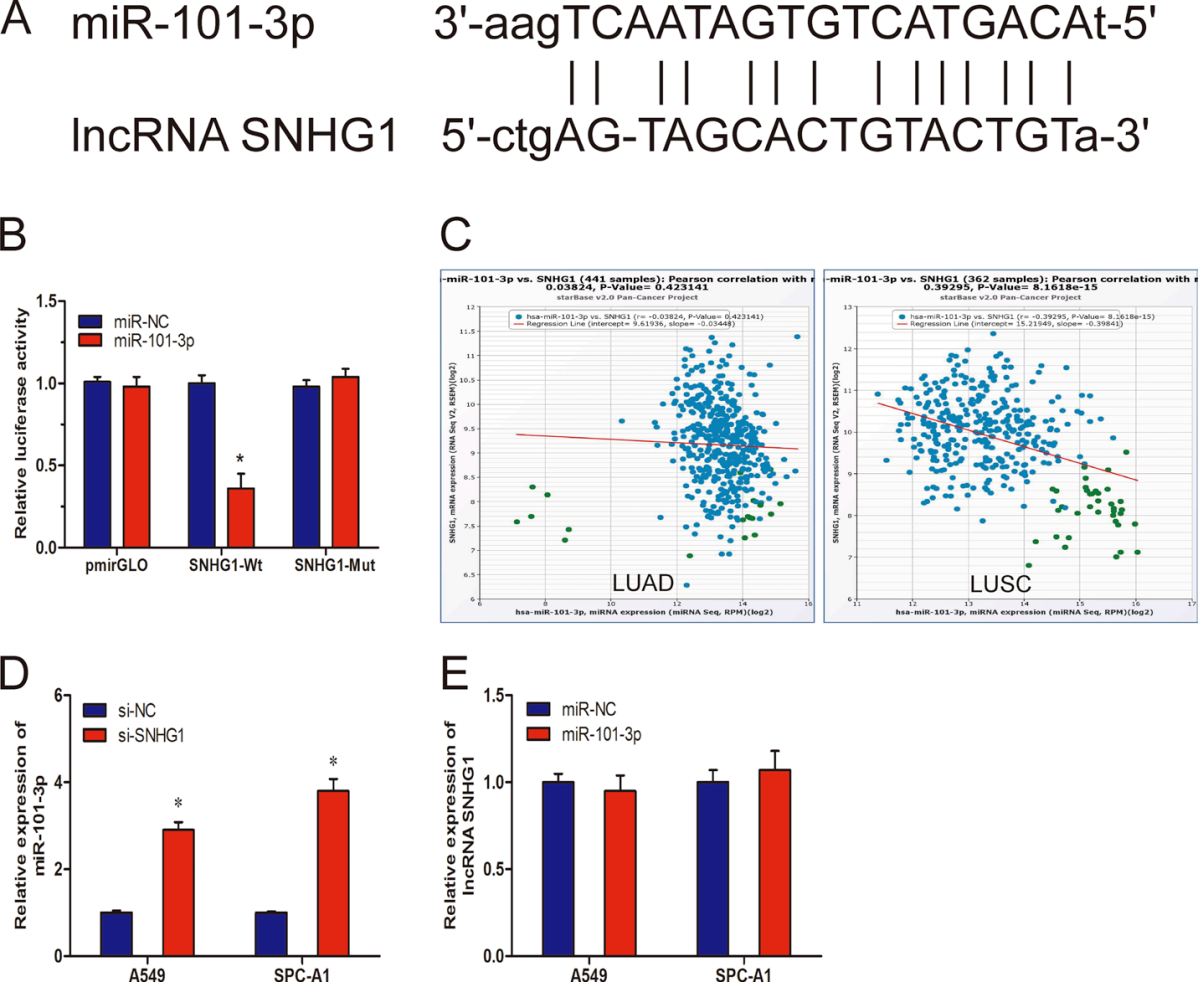
LncRNA SNHG1 directly interacted with miR-101-3p (**A**) Sequence alignment of miR-101-3p with the putative binding sites with in the wild-type regions of SNHG1. (**B**) Dual-luciferase reporter assay showed that miR-101-3p mimics reduced the intensity of fluorescence in HEK293T cells transfected with SNHG1-Wt, while had no effect on the SNHG1-mut vector. (**C**) The correlation between SNHG1 mRNA and miR-101-3p expression in NSCLC tissues. (**D**) qRT-PCR analysis of miR-101-3p expression in A549 and SPC-A1 cells transfected with si-SNHG1 or si-NC. (**E**) qRT-PCR analysis of SNHG1 expression in A549 and SPC-A1 cells transfected with miR-101-3p or miR-NC. *P < 0.05.

Recently, mounting evidence showed that lncRNAs contain motif with sequence complementary to miRNAs and have an inhibition effect on miRNAs expression and activity [13, 14]. To examine whether SNHG1 has a similar mechanism in NSCLC, prediction of miRNA target sites was performed by the online software starBase v2.0 (Figure 4A). Ddual-luciferase reporter assay was performed to explore whether SNHG1 was a functional target of miR-101-3p. We found that the luciferase activity was significantly decreased by the co-transfection of miR-101-3p mimics and SNHG1-Wt rather than the co-transfection of miR-NC and SNHG1-Wt, meanwhile, co-transfection of miR-101-3p mimics and SNHG1-Mut did not change the luciferase activity (Figure 4B). To further support this conclusion, we explored the association between SNHG1 and miR-101-3p expression from TCGA Data Portal, we found that SNHG1 expression was correlated with miR-101-3p expression in NSCLC tissues (Figure 4C). In addition, we found that inhibition of SNHG1 by siRNA led to significantly increased expression of miR-101-3p (Figure 4D; *P* < 0.05). However, up-regulated expression of miR-101-3p was not able to affect SNHG1 expression (Figure 4E; *P* > 0.05). Thus, these results indicated that miR-101-3p was an inhibitory target for SNHG1 in NSCLC progression.

### SOX9 is a target gene of miR-101-3p and is regulated by SNHG1

**Figure 5:**
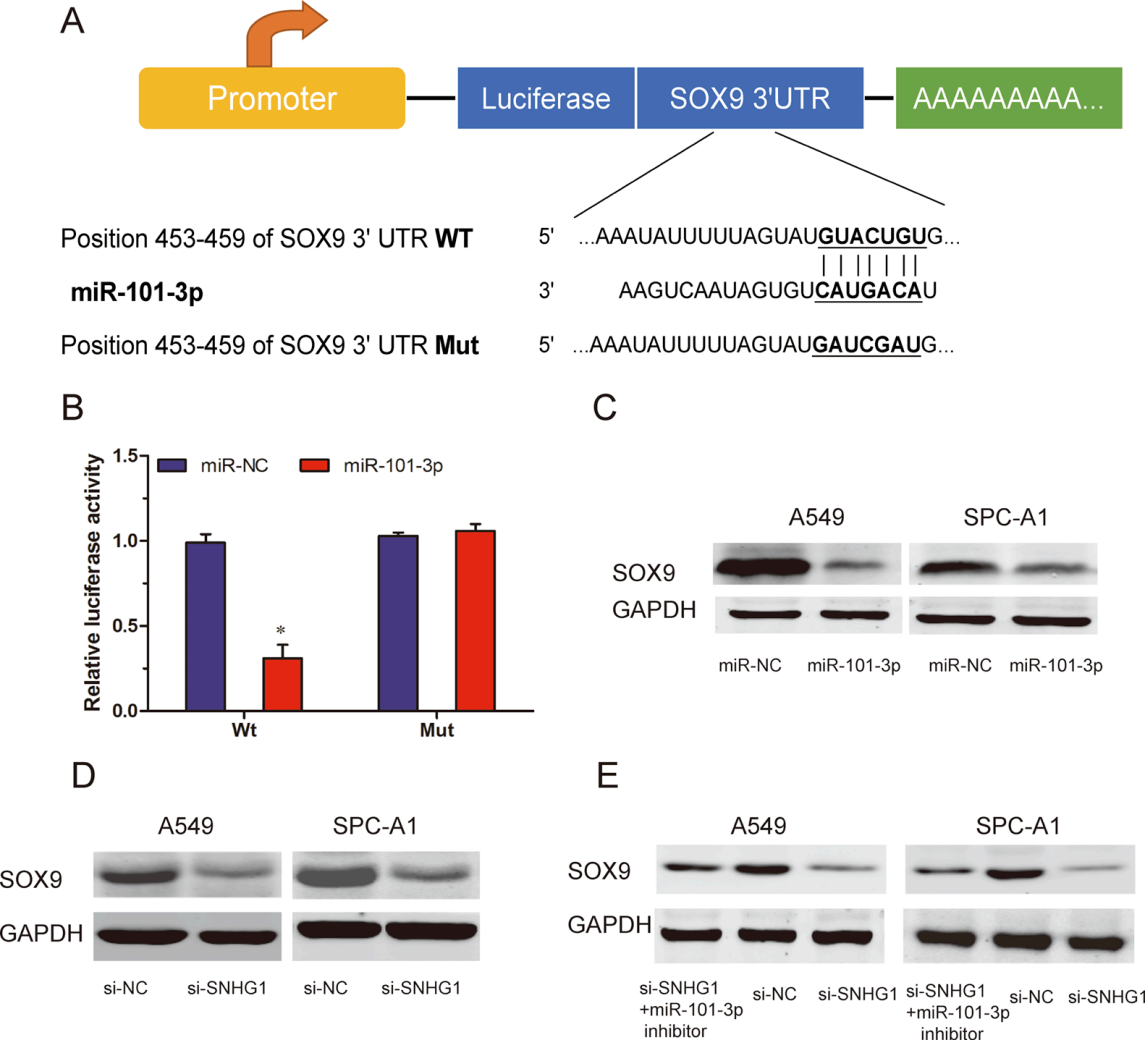
SOX9 is a target of miR-101-3p and is suppressed by SNHG1 inhibition (**A**) A schematic illustration of wt and Mut 3′-UTR of SOX9. (**B**) Dual-luciferase reporter assay revealed that miR-101-3p inhibited wt SOX9 3′-UTR luciferase activity, while it had no effect on Mut SOX9 3′-UTR luciferase activity in HEK293T cells. (**C**) Expression of SOX9 was determined by western blot in A549 and SPC-A1 cells transfected with miR-101-3p mimics or miR-NC. (**D**) Protein levels of SOX9 in A549 and SPC-A1 cells transfected with si-SNHG1 or si-NC explored by Western blot. (**E**) Protein levels of SOX9 expression in A549 and SPC-A1 cells transfected with si-SNHG1 or si-NC in the presence or absence of miR-101-3p inhibitor. *P < 0.05.

We used TargetScan to predict target genes for miR-101-3p, and SOX9 came out as one of the best candidates (Figure 5A). In order to confirm the prediction, we first constructed luciferase reporter plasmids harboring either the wild-type 3′-UTR of SOX9 or a mutant 3′-UTR predicted to be insensitive to miR-101-3p. We transfected HEK293T cells with the luciferase reporter plasmids, together with miR-101-3p mimics. Our data showed that miR-101-3p overexpression significantly reduced the luciferase activity of the wild-type 3′-UTR of SNHG1, but not the mutant 3′-UTR of SOX9 in HEK293T cells (Figure 5B; *P* < 0.05). To further confirm the regulation of SOX9 by miR-101-3p, we determined examined the protein expression of SOX9 when miR-101-3p was overexpressed. Our results showed that increased expression of miR-101-3p markedly decreased the protein levels of SOX9 (Figure 5C; *P* < 0.05). These findings suggested that SOX9 was a target gene of miR-101-3p.

To further explore whether SOX9 was regulated by SNHG1. In A549 and SPC-A1 cells, our data showed that inhibition of SNHG1 reduced the protein levels of SOX9 (Figure 5D; *P* < 0.05). Furthermore, we found that knockdown of SNHG1 was no longer able to reduce SOX9 protein expression when miR-101-3p was suppressed (Figure 5E; *P* < 0.05). Thus, these results suggested that the regulation of SOX9 by SNHG1 required the activity of miR-101-3p.

### SOX9 activates the Wnt/β-catenin signaling pathways

**Figure 6:**
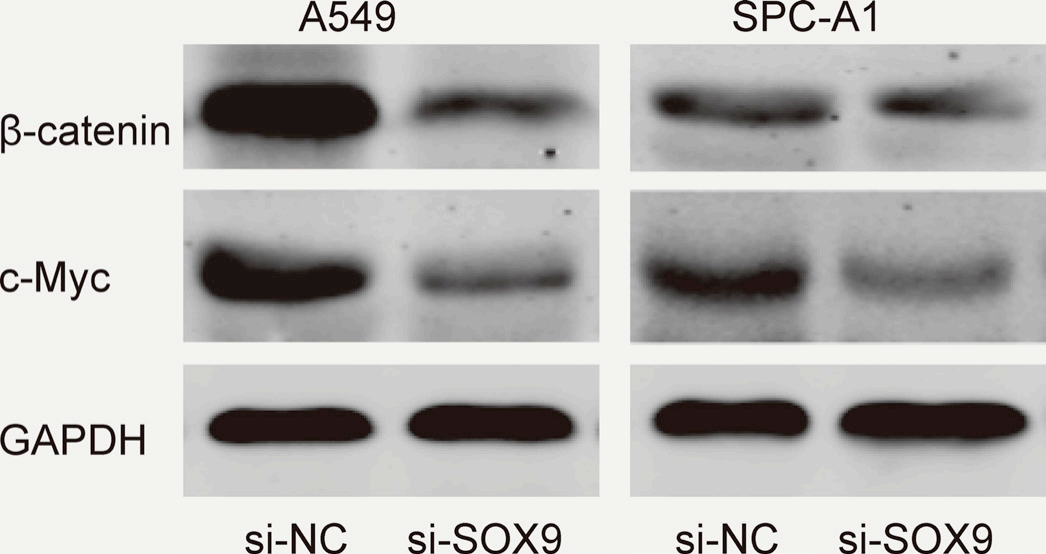
SOX9 activated the Wnt/β-catenin signaling pathway in A549 and SPC-A1 cells

**Figure 7:**
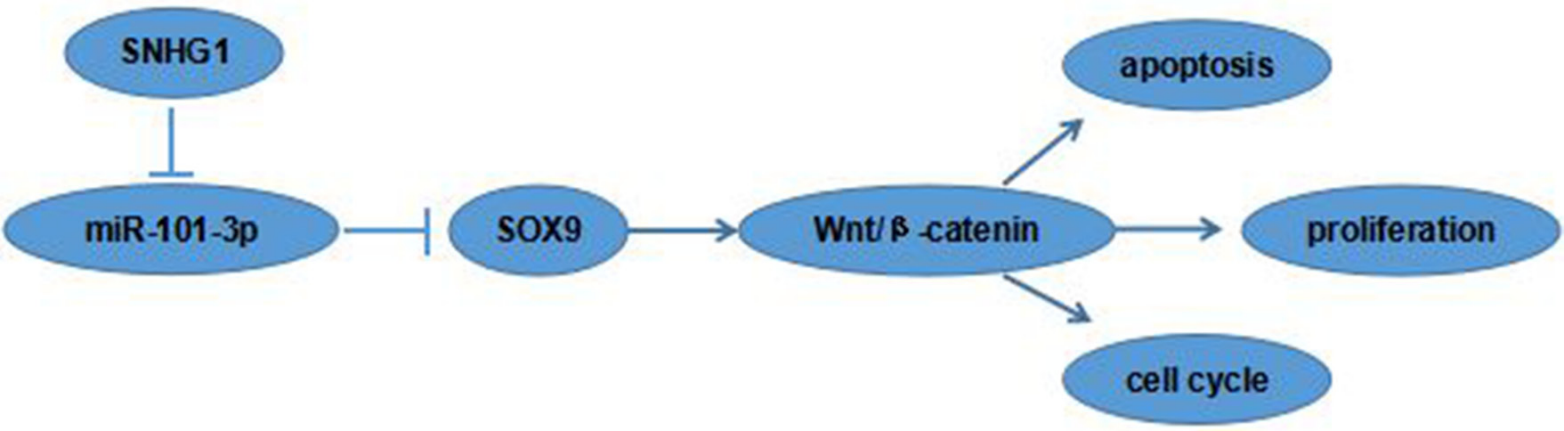
The cartoon of the mechanism underlying the SNHG1-miR-101-3p-SOX9 axis in NSCLC

Recent studies showed that SOX9 playing crucial roles in cancer cell proliferation, migration, angiogenesis and survival [15]. To figure out the molecular mechanisms of SOX9 oncogenic functions, the activities of Wnt/β-catenin signaling pathway was detected in the A549 and SPC-A1 cell lines. Western blot showed that the protein expression levels of β-catenin and c-Myc were markedly reduced in the si-SOX9 transfected A549 cells compared to si-NC group (Figure 6), indicating Wnt/β-catenin signaling pathway was involved in SOX9 oncogenic functions. Our data suggested that the SNHG1/miR-101-3p /SOX9/Wnt/β-catenin axis play an important role in NSCLC progression and development (Figure 7).

## DISCUSSION

Accumulating evidence suggested that lncRNAs were associated with tumor invasiveness and metastatic potential, and were thought of as being diagnostic and prognostic markers for several types of cancer, including lung cancer [16]. For example, Chen et al. showed that low expression lncRNA TUBA4B was a poor predictor of prognosis and regulated cell proliferation in NSCLC [17]. Nie et al. found that lncRNA ANRIL promoted NSCLC cell proliferation and inhibited apoptosis by silencing KLF2 and P21 expression [18]. However, the function and underlying mechanism of lncRNAs in NSCLC remain unclear.

Recent reports showed that SNHG1 play important roles in tumor progression. For example, Zhang et al. showed that SNHG1 was up-regulated in hepatocellular carcinoma (HCC) and correlated with HCC progression, furthermore, they found that SNHG1 promoted HCC cells proliferation through inhibiting p53 and p53-target genes expression [19]. Sahu et al. indicated that SNHG1 acted as a novel predictor for event-free survival in neuroblastoma [20]. Recently, You et al. showed that SNHG1 could promote cell proliferation in NSCLC [21]. However, the clinical significance and underlying mechanism are still unclear.

In the present study, we found that SNHG1 expression in NSCLC tissues was significantly increased compared to adjacent non-tumor tissues. High expression of SNHG1 in NSCLC patients was associated with larger tumor size, advanced TNM stage, lymph node metastasis and poor overall survival. Furthermore, we explored the biological function of SNHG1 in NSCLC. Our results showed that SNHG1 inhibition markedly suppressed NSCLC cell proliferation both *in vitro* and *in vivo*. In addition, flow cytometric analysis revealed that the inhibitory effect of SNHG1 on NSCLC cell proliferation by causing G1/G0 phases arrest and inducing cell apoptosis. These data suggested that SNHG1 acted as a tumor oncogene in NSCLC progression.

In recent years, lncRNA has been shown to be a sponge for regulating the expression and activity of miRNA [13]. For example, Shao et al. reported that lncRNA RMRP promoted carcinogenesis by acting as a miR-206 sponge and is used as a novel biomarker for gastric cancer [22]. Li et al. suggested that lncRNA UCA1 promoted glutamine metabolism by targeting miR-16 in human bladder cancer [23]. Liu et al. suggested that lncRNA RSU1P2 contributed to tumorigenesis by acting as a ceRNA against let-7a in cervical cancer cells [24]. However, limited knowledge is available concerning the relationship between lncRNA and miRNA in the carcinogenesis of NSCLC, which needs to be well documented. In this study, we identified miR-101-3p as an inhibitory target of SNHG1 by sequence complementarity analysis and luciferase reporter assay. Pearson correlation analysis showed a remarkably negative correlation between SNHG1 expression and miR-101-3p in NSCLC tissues. In addition, reduction of SNHG1 expression increased miR-101-3p expression and increased miR-101-3p expression was not able to affect the SNHG1 expression. Thus, these results suggested that miR-101-3p is a direct target of SNHG1.

Wnt/β-catenin signaling is known to regulate a broad range of cellular processes, such as proliferation, invasion, differentiation and other signaling pathways, through regulating the ability of the multifunctional β-catenin protein, which is a crucial signaling molecule in the Wnt/β-catenin pathway [25, 26]. Recent studies showed a significant correlation between Wnt/β-catenin signaling pathway and SOX9 in tumor progression. For example, Santos et al. found that SOX9 elevation acted with canonical Wnt signaling to drive gastric cancer progression [27]. Ma et al. reported that SOX9 drived Wnt pathway activation in prostate cancer [28]. Prévostel et al. showed that SOX9 was an atypical intestinal tumor suppressor controlling the oncogenic Wnt/ß-catenin signaling [29]. In our study, we identified that SOX9 could act as a target of miR-101-3p, and found that SOX9 played the oncogenic role in NSCLC progression by activating Wnt/β-catenin signaling pathway. Our study expanded the function of SOX9 in NSCLC progression.

In conclusion, our study showed that SNHG1 act as an oncogenic lncRNA that promoted NSCLC tumorigenesis and progression via miR-101-3p/SOX9/Wnt/β-catenin axis. Therefore, these findings suggested that lncRNA SNHG1 could act as a potential therapeutic target for the treatment of NSCLC.

## MATERIALS AND METHODS

### Cell culture

Four NSCLC cell lines (A549, SPC-A1, H23 and NCI-H520) and human bronchial epithelial cell line (16HBE) were purchased from the Institute of Biochemistry and Cell Biology of the Chinese Academy of Sciences (Shanghai, China). Cells were maintained in RPMI-1640 with 10% fetal bovine serum (FBS, GIBCO), 100 U/ml penicillin and 100 mg/ml streptomycin in humidified air at 37°C with 5% CO_2_.

### Tissue samples collection

Sixty eight paired NSCLC tissues and adjacent non-tumor tissues were obtained from patients who had undergone surgery at Henan Provincial Cancer Hospital, between 2010 and 2011 and who were diagnosed with NSCLC based on histopathological evaluation. No local or systemic treatment had been conducted in these patients before the operation. All the tissue samples were collected, immediately snap frozen in liquid nitrogen, and stored at −80°C until RNA extraction. The study was approved by the Research Ethics Committee of Henan Provincial Cancer Hospital. Informed consent was obtained from all patients.

### Cell transfection

The small interfering RNAs (siRNAs) specifically targeting SNHG1 were purchased from Invitrogen. The miR-101-3p mimics and miR-101-3p inhibitors were purchased from Guangzhou RiboBio Co. Ltd. The siRNA sequences for SNHG1 were si-SNHG1, 5′-CAGCAGTTGAGGGTTTGCTGTGTAT-3′ [19]. NSCLC cells were transfected with siRNA oligonucleotides, miR-101-3p mimics or miR-101-3p inhibitors using Lipofectamine 2000 (Invitrogen), according to the manufacturer's protocol.

### Cell proliferation assay

Cell proliferation was measured using the cell proliferation reagent WST-8 (Roche Biochemicals). After plating cells in 96-well microtiter plates (Corning Costar) at 1.0 × 10^3^/well, 10 μl of CCK-8 was added to each well at the time of harvest, according to the manufacturer's instructions. 2 h after adding CCK-8, cellular viability was determined by measuring the absorbance of the converted dye at 450 nm.

### Cell cycle analysis

The transfected cells were harvested and then fixed with 500 μl of 70% cold ethanol for 2 h. The cells were added with 100 μl of RNase and incubated at 37°C for 30 min. Then, 400 μl of PI was added, and the cells were incubated at 4°C for 30 min away from light. The samples were immediately subjected to flow cytometer (FACScan, BD Biosciences). The results were analyzed using CELL Quest 3.0 software.

### Cell apoptosis analysis

The transfected cells were harvested after transfection by trypsinization. After the double staining with FITC-Annexin V and PI was done by the FITC-Annexin V Apoptosis Detection Kit (BD Biosciences) according to the manufacturer's protocol. The cells were analyzed with a flow cytometry (FACScan, BD Biosciences) equipped with a Cell Quest 3.0 software. Cells were discriminated into viable cells, dead cells, early apoptotic cells, and apoptotic cells.

### Tumor formation assay in a nude mouse model

5-week-old female athymic BALB/c mice were maintained under specific pathogen-free conditions and manipulated according to protocols approved by the Shanghai Medical Experimental Animal Care Commission. A549 cells transfected with sh-SNHG1 or sh-NC were harvested at a concentration of 2 × 10^7^ cells/ml. Of the suspending cells, 0.1 ml was subcutaneously injected into either side of the posterior flank of the nude mouse. Tumor volumes were examined every 7 days when the implantations were starting to grow bigger. Tumor wights were measured. All mice were killed 49 days after injection and tumors were weighed.

### Reporter vectors construction and luciferase reporter assay

The fragment from SNHG1 containing the predicted miR-101-3p binding site was amplified by PCR and then cloned into a pmirGlO Dual-luciferase miRNA Target Expression Vector (Promega) to form the reporter vector SNHG1-wild-type (SNHG1-Wt). To mutate the putative binding site of miR-101-3p in the SNHG1, the sequence of putative binding site was replaced as indicated and was named as SNHG1-mutated-type (SNHG1-Mut). Similarly, the fragment from SOX9 3′-UTR sequences were amplified by PCR and cloned into a pmirGlo Dual-luciferase miRNA Target Expression Vector to form the reporter vector SOX9-wild-type (SOX9-Wt) (GenePharma). To mutate the putative binding site of miR-101-3p in the 3′-UTR containing vector, the sequence of putative binding site was replaced as indicated and was named as SOX9-mutated-type (SOX9-Mut). Then the vectors and miR-101-3p mimics were co-transfected into HEK293T cells, and the Dual-luciferase reporter assay system (Promega) was used for testing the luciferase activity.

### Quantitative realtime-PCR

Total RNA was extracted from tissues or cultured cells using TRIzol reagent (Invitrogen). For qRT-PCR, RNA was reverse transcribed to cDNA by using a Reverse Transcription Kit (Takara). Real-time PCR analysis was performed with SYBR Green (Takara). Results were normalized to the expression of GAPDH (for lncRNAs) or U6 (for miRNAs). QRT-PCR reactions were performed by the ABI7500 system (Applied Biosystems). The relative expression fold change of mRNA was calculated by the 2^−ΔΔCt^ method. The primer sequences were as follows: SNHG1, forward: 5′-AGGCTGAAGTTACAGGTC-3′ and reverse: 5′-TTGGCTCCCAGTGTCTTA-3′; GAPDH, forward: 5′-GTCAACGGATTTGGTCTGTATT-3′ and reverse: 5′-AGTCTTCTGGGTGGCAGTGAT-3′. MiR- 101-3p, forward: 5′-UACAGUACUGUGAUAACUGA A-3′ and reverse: 5′-CAGUUAUCACAGUACUGUAU U-3′; U6, forward: 5′-GCUUCGGCAGCACAUAUACUA AAAU-3′ and reverse: 5′-CGCUUCACGAAUUUGCGU GUCAU-3′.

### Western blot

Cells were lysed using RIPA protein extraction reagent (Beyotime) supplemented with PMSF (Riche). Approximately 25 μg of protein extracts were separated by 10% sodium dodecyl sulfate polyacrylamide gel electrophoresis (SDS-PAGE), transferred onto nitrocellulose membranes (Sigma), and were probed with primary antibodies against SOX9, β-catenin, c-myc (Abcam) or GAPDH (Abcam). Membranes were incubated at 4°C overnight, followed by incubation with AP-conjugated secondary antibodies and detected by enhanced chemiluminescent (ECL).

### Statistical analysis

All statistical analyses were performed using SPSS 20.0 software. Data was presented as mean ± SD. The significance of differences between groups were estimated by Student's *t*-test, χ^2^ test or Wilcoxon test as appropriate. Cumulative overall survival was evaluated by Kaplan-Meier method with the log-rank test applied for comparison. *P* < 0.05 was considered statistically significant.

## References

[1] Jemal A, Bray F, Center MM, Ferlay J, Ward E, Forman D (2011). Global cancer statistics. CA Cancer J Clin.

[2] Cruz CSD, Tanoue LT, Matthay RA (2011). Lung cancer: epidemiology, etiology, and prevention. Clinics in chest medicine.

[3] Goldstraw P, Ball D, Jett JR, Le Chevalier T, Lim E, Nicholson AG, Shepherd FA (2011). Non-small-cell lung cancer. Lancet.

[4] Rosell R, Bivona TG, Karachaliou N (2013). Genetics and biomarkers in personalisation of lung cancer treatment. Lancet.

[5] Peters S, Adjei AA, Gridelli C, Reck M, Kerr K, Felip E (2012). Metastatic non-small-cell lung cancer (NSCLC): ESMO Clinical Practice Guidelines for diagnosis, treatment and follow-up. Ann Oncol.

[6] Mercer TR, Dinger ME, Mattick JS (2009). Long non-coding RNAs: insights into functions. Nature Reviews Genetics.

[7] Fatica A, Bozzoni I (2014). Long non-coding RNAs: new players in cell differentiation and development. Nat Rev Genet.

[8] Qureshi IA, Mattick JS, Mehler MF (2010). Long non-coding RNAs in nervous system function and disease. Brain Res.

[9] Gibb EA, Brown CJ, Lam WL (2011). The functional role of long non-coding RNA in human carcinomas. Mol Cancer.

[10] Zhang HM, Yang FQ, Chen SJ, Che J, Zheng JH (2015). Upregulation of long non-coding RNA MALAT1 correlates with tumor progression and poor prognosis in clear cell renal cell carcinoma. Tumour Biol.

[11] Li R, Zhang L, Jia L, Duan Y, Li Y, Bao L, Sha N (2014). Long non-coding RNA BANCR promotes proliferation in malignant melanoma by regulating MAPK pathway activation. PLoS One.

[12] Ke J, Yao YL, Zheng J, Wang P, Liu YH, Ma J, Li Z, Liu XB, Li ZQ, Wang ZH, Xue YX (2015). Knockdown of long non-coding RNA HOTAIR inhibits malignant biological behaviors of human glioma cells via modulation of miR-326. Oncotarget.

[13] Jalali S, Bhartiya D, Lalwani MK, Sivasubbu S, Scaria V (2013). Systematic transcriptome wide analysis of lncRNA-miRNA interactions. PLoS One.

[14] Paraskevopoulou MD, Georgakilas G, Kostoulas N, Reczko M, Maragkakis M, Dalamagas TM, Hatzigeorgiou AG (2013). DIANA-LncBase: experimentally verified and computationally predicted microRNA targets on long non-coding RNAs. Nucleic Acids Res.

[15] Lu B, Fang Y, Xu J, Wang L, Xu F, Xu E, Huang Q, Lai M (2008). Analysis of SOX9 expression in colorectal cancer. Am J Clin Pathol.

[16] Prensner JR, Chinnaiyan AM (2011). The emergence of lncRNAs in cancer biology. Cancer Discov.

[17] Chen J, Hu L, Wang J, Zhang F, Chen J, Xu G, Wang Y, Pan Q (2016). Low Expression LncRNA TUBA4B is a Poor Predictor of Prognosis and Regulates Cell Proliferation in Non-Small Cell Lung Cancer. Pathology & Oncology Research.

[18] Nie FQ, Sun M, Yang JS, Xie M, Xu TP, Xia R, Liu YW, Liu XH, Zhang EB, Lu KH, Shu YQ (2015). Long noncoding RNA ANRIL promotes non-small cell lung cancer cell proliferation and inhibits apoptosis by silencing KLF2 and P21 expression. Mol Cancer Ther.

[19] Zhang M, Wang W, Li T, Yu X, Zhu Y, Ding F, Li D, Yang T (2016). Long noncoding RNA SNHG1 predicts a poor prognosis and promotes hepatocellular carcinoma tumorigenesis. Biomed Pharmacother.

[20] Sahu D, Hsu C-L, Lin C-C, Yang T-W, Hsu W-M, Ho S-Y, Juan H-F, Huang H-C (2016). Co-expression analysis identifies long noncoding RNA SNHG1 as a novel predictor for event-free survival in neuroblastoma. Oncotarget.

[21] You J, Fang N, Gu J, Zhang Y, Li X, Zu L, Zhou Q (2014). Noncoding RNA small nucleolar RNA host gene 1 promote cell proliferation in nonsmall cell lung cancer. Indian J Cancer.

[22] Shao Y, Ye M, Li Q, Sun W, Ye G, Zhang X, Yang Y, Xiao B, Guo J LncRNA-RMRP promotes carcinogenesis by acting as a miR-206 sponge and is used as a novel biomarker for gastric cancer. Oncotarget.

[23] Li HJ, Li X, Pang H, Pan JJ, Xie XJ, Chen W (2015). Long non-coding RNA UCA1 promotes glutamine metabolism by targeting miR-16 in human bladder cancer. Jpn J Clin Oncol.

[24] Liu Q, Guo X, Que S, Yang X, Fan H, Liu M, Li X, Tang H (2016). LncRNA RSU1P2 contributes to tumorigenesis by acting as a ceRNA against let-7a in cervical cancer cells. Oncotarget.

[25] Cadigan KM (2008). Wnt–β-catenin signaling. Current Biology.

[26] Clevers H, Nusse R (2012). Wnt/beta-catenin signaling and disease. Cell.

[27] Santos JC, Carrasco-Garcia E, Garcia-Puga M, Aldaz P, Montes M, Fernandez-Reyes M, de Oliveira CC, Lawrie CH, Arauzo-Bravo MJ, Ribeiro ML, Matheu A (2016). SOX9 Elevation Acts with Canonical WNT Signaling to Drive Gastric Cancer Progression. Cancer Res.

[28] Ma F, Ye H, He HH, Gerrin SJ, Chen S, Tanenbaum BA, Cai C, Sowalsky AG, He L, Wang H, Balk SP, Yuan X (2016). SOX9 drives WNT pathway activation in prostate cancer. J Clin Invest.

[29] Prévostel C, Rammah-Bouazza C, Trauchessec H, Canterel-Thouennon L, Busson M, Ychou M, Blache P (2016). SOX9 is an atypical intestinal tumor suppressor controlling the oncogenic Wnt/ß-catenin signaling. Oncotarget.

